# New Drugs, Appliances, and Things Medical

**Published:** 1890-01-18

**Authors:** 


### NEW DRUGS, APPLIANCES AND THINGS MEDICAL.

[All preparations, appliances, novelties, etc., of which a notice is desired, should be sent to The Editor, The Lodge, Porchester Square, W.]

### STERN'S PUMILINE PREPARATIONS.

Amongst these we notice an essence, ointment, liniment, superfatted soap, and other forms for administration as jujubes and as extract for baths. Pumiline essence is the active principle distilled from the needles of the snowgrown pinus pumilio. It is a light, amber coloured, clear fluid with an agreeable pine-like odour. It has been combined with suitable bases in different forms both for internal and external medication. We can speak decidedly in favour of these. The extract for use in baths, more especially hot baths, has proved in our hands most beneficial in relieving the pains in the limbs during an attack of the prevailing epidemic, leaving the patient decidedly easier and much refreshed. The liniment is useful for external application in similar cases, and the essence administered internally in the form of vapour, in some cases, where the bronchial symptoms were prominent, gave great relief. The soap is a very good one for delicate skins, as it distinctly mitigates the harshness produced by London water; and being superfatted it causes less irritation than more alkaline forms of soap. The ointment is a pleasant preparation to be used where an antiseptic, soothing, non-irritant form of greasy application is required. We have no doubt that these pleasant forms of terebinthine remedies will have considerable success.

### SCIANDRA'S PRESERVED NATURAL PULQUE.

We have received a specimen of this, which is the preserved juice of the American century plant, from Mr. James Clark, North Bruntsfield-place, Edinburgh, the sole importer. It is a milky-looking fluid, acid to test paper, containing a fair amount of alcohol resulting from the natural fermentation the product undergoes. It has an odour somewhat resembling cider. On tasting it, it is perceived to be distinctly astringent in flavour, which is not due to tannin, however, as iron solutions have no colour reaction with it. It is free from sugar, both added and natural. There is a slight precipitate, which the directions state should be well shaken up before using. According to the testimonials accompanying the specimen, the preparation has been successfully used in Bright's disease, reducing the albumen, and even effecting a cure. It has also been used with success in dyspepsia, with constipation, and in chronic leucorrhœa. A very interesting pamphlet accompanies the preparation describing the use of pulque by the Mexicans, both as a food-drink and as a medicine. The pulque seems to have been used from the earliest times, the Spaniards acquiring the habit of drinking it from the Aztecs. The company which sends out this preparation states that by a simple process it stops the fermentation at the right point, and so prevents the liquid from becoming useless by over-development of acid. We may add that it is not a patent medicine.

